# Audiovisual simultaneity windows reflect temporal sensory uncertainty

**DOI:** 10.3758/s13423-024-02478-4

**Published:** 2024-02-22

**Authors:** Emma Cary, Ilona Lahdesmaki, Stephanie Badde

**Affiliations:** Department of Psychology, Tufts University, Medford, MA 02155 USA

**Keywords:** Visual, Auditory, Temporal, Perception, Multisensory integration, Simultaneity judgment, Uncertainty, Ideal observer models, Decision criteria

## Abstract

The ability to judge the temporal alignment of visual and auditory information is a prerequisite for multisensory integration and segregation. However, each temporal measurement is subject to error. Thus, when judging whether a visual and auditory stimulus were presented simultaneously, observers must rely on a subjective decision boundary to distinguish between measurement error and truly misaligned audiovisual signals. Here, we tested whether these decision boundaries are relaxed with increasing temporal sensory uncertainty, i.e., whether participants make the same type of adjustment an ideal observer would make. Participants judged the simultaneity of audiovisual stimulus pairs with varying temporal offset, while being immersed in different virtual environments. To obtain estimates of participants’ temporal sensory uncertainty and simultaneity criteria in each environment, an independent-channels model was fitted to their simultaneity judgments. In two experiments, participants’ simultaneity decision boundaries were predicted by their temporal uncertainty, which varied unsystematically with the environment. Hence, observers used a flexibly updated estimate of their own audiovisual temporal uncertainty to establish subjective criteria of simultaneity. This finding implies that, under typical circumstances, audiovisual simultaneity windows reflect an observer’s cross-modal temporal uncertainty.

**Supplementary Information:**

The online version contains supplementary material available at 10.3758/s13423-024-02478-4.

## Introduction

Integrating information from vision and audition provides clear perceptual advantages, as many of us realized when trying to order a coffee while wearing a face mask. However, integrating visual and auditory signals from different sources, such as the lip movements of a character on TV and the voice of your partner talking to you, may lead to incorrect perceptual decisions. One important indicator of whether visual and auditory signals belong together is their temporal relation. Simultaneous visual and auditory signals might originate from the same source, while a light impression and a sound that occur with a large temporal offset are unlikely to provide information about the same event. Yet, each temporal measurement is associated with error due to noise in the environment and the stochasticity of the neuronal system. This measurement error is variable and best accounted for by allowing for some offset when categorizing two sensory signals as temporally aligned or misaligned. Indeed, cross-modal stimulus pairs presented with a small temporal offset between them are integrated (Alais et al., 2010; Colonius & Diederich, 2004; Slutsky & Recanzone, 2001; Van Atteveldt et al., 2006), and multisensory integration effects decrease gradually with increasing temporal discrepancy between the stimuli (Koppen & Spence, 2007; Lewald et al., 2001; Van Wassenhove et al., 2007).

The most popular tool to measure an observer’s tolerance for cross-modal asynchrony are simultaneity judgments (Fujisaki et al., 2012). In this task, participants are presented with audiovisual stimulus pairs with variable temporal offsets and indicate for each pair whether they perceived it as simultaneous or not. The range of temporal offsets that are reliably perceived as simultaneous is called the audiovisual simultaneity (Roseboom et al., 2009) or “binding” window (Stevenson & Wallace, 2013; Wallace & Stevenson, 2014). The advantages of this task are its simplicity, which renders it ideal for research with special populations, as well as the relatively low number of trials needed to establish the width of the simultaneity window (Wallace & Stevenson, 2014). A large body of research has assessed audiovisual simultaneity windows across different stimulus types (Eg & Behne, 2015; Horsfall et al., 2021; Leone & McCourt, 2015; Roseboom et al., 2009; Stevenson & Wallace, 2013; Van Eijk et al., 2008; Vroomen & Keetels, 2020; Wallace & Stevenson, 2014; Zampini et al., 2005) and modality combinations (Machulla et al., 2016) in typically developed individuals of all ages (Basharat et al., 2018; Chen et al., 2016; Hillock et al., 2011; Hillock-Dunn & Wallace, 2012; Noel et al., 2016) as well as individuals with neurodevelopmental disorders (Donohue et al., 2012; Panagiotidi et al., 2017; Stevenson et al., 2014), in persons with sensory impairments (Peter et al., 2019; Schormans & Allman, 2018; Shayman et al., 2018; Stevenson, Park, et al., 2017a, Stevenson, Sheffield, et al., 2017b), and in animals (Schormans et al., 2017).

**Fig. 1:**
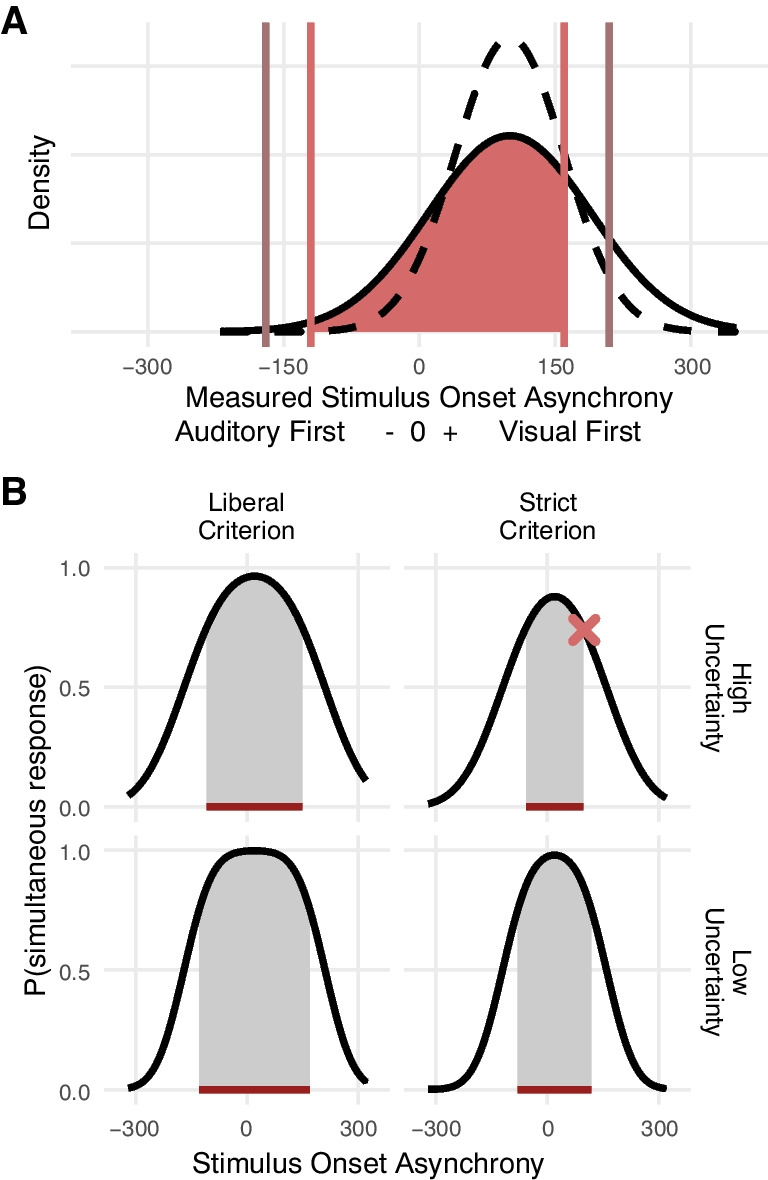
Audiovisual simultaneity perception depends on temporal uncertainty and subjective decision criteria. (**A**) Each temporal measurement is associated with random error. The figure shows two probability density functions of measured audiovisual temporal offsets (negative values indicate that the auditory stimulus was registered first) for a situation in which the visual stimulus led the auditory one by 100 ms. The probability distribution varies with the observer’s temporal uncertainty (here, parametrized as standard deviation of a Gaussian distribution; solid line: 90 ms, dashed line: 60 ms). We assume an observer categorizes a measured temporal offset as a measurement error or as evidence for asynchronous stimuli by comparing the measurement to subjective boundaries. These boundaries might differ with the direction of the measured offset (auditory or visual first); they might be close to each other (a strict simultaneity criterion; salmon vertical lines), or more spread out (a liberal criterion; taupe vertical lines). The shaded area equals the probability to judge a stimulus pair with an onset asynchrony of 100 ms as simultaneous given high temporal uncertainty and strict criteria (see salmon-colored marker in B). (**B**) The experimenter has no direct access to the processes within the observer’s brain depicted in (A) but can infer them from the observer’s behavior. For example, the salmon-colored cross in (B) indicates the probability of a “simultaneous”-judgment for a stimulus-onset asynchrony of 100 ms (visual first) given high uncertainty and strict criteria, the probability corresponds to the salmon-shaded region in (A). The simultaneity window is the range of presented audiovisual temporal offsets for which stimulus pairs are likely to be perceived as simultaneous (dark red horizontal line; here, a probability of 75% was used to define the window). The width of the simultaneity window depends on both the observer’s audiovisual temporal uncertainty (rows) as well as their subjective criteria of simultaneity (columns). Thereby, the effect of temporal uncertainty declines when a lower probability of “simultaneous”-responses is used to define the simultaneity window, whereas changes in the criterion lead to definition-independent changes in the simultaneity window

The width of the audiovisual window of simultaneity is determined by two factors: (1) an observer’s audiovisual temporal uncertainty and (2) their subjective simultaneity criteria or decision boundaries (Fig. 1). (1) With increasing temporal uncertainty, the probability of large temporal measurement errors increases (Fig. 1A, solid vs. dashed line). Hence, with increasing temporal uncertainty, simultaneously presented stimuli are less likely to be perceived as simultaneous (Fig. 1B, top vs. bottom row). (2) The boundary between a measured temporal offset small enough to reflect only measurement error and measured offsets that indicate temporally misaligned sensory signals is determined by the observer. Thus, an observer could be strict and set narrow boundaries or be liberal and allow for larger temporal offsets (Fig. 1A; salmon vs. taupe vertical lines). The more liberal the subjective simultaneity criteria are, the wider the window of audiovisual simultaneity is (Fig. 1B; left vs. right column).

Here, we investigated the relationship between the two determinants of audiovisual simultaneity windows: audiovisual temporal uncertainty and subjective simultaneity judgment criteria. Specifically, we tested the hypothesis that participants will employ an approach similar to that of ideal observers and flexibly set the decision boundaries based on their situational temporal uncertainty. The intuition behind this hypothesis is simple: The larger the expected temporal measurement error, the more permissive an observer should set the criteria that account for such measurement errors.

Previous studies provide only indirect evidence about the influence of temporal uncertainty on audiovisual simultaneity judgments. On one hand, simultaneity windows are influenced by explicit instructions about the criterion (Yarrow et al., 2023) and subjective decision biases (Linares et al., 2019). These results underline that simultaneity judgments rely on subjective criteria and thus support the notion that simultaneity windows are an ambiguous measure of cross-modal temporal processing (Yarrow et al., 2011). On the other hand, simultaneity windows vary with many factors that influence sensory uncertainty such as age (Noel et al., 2016) or the presence and timing of concurrent movements (Arikan et al., 2017; Benedetto et al., 2018). Moreover, the width of simultaneity windows correlates with variables that are strongly influenced by sensory uncertainty, such as one’s susceptibility to sensory illusions (Costantini et al., 2016; Stevenson et al., 2012) or propensity for short-term recalibration (Noel et al., 2016). Finally, perceptual training, which should lead to a decrease in temporal uncertainty, narrows audiovisual simultaneity windows (De Niear et al., 2018; Lee & Noppeney, 2011; McGovern et al., 2016, 2022; Powers et al., 2009, 2012; Stevenson et al., 2013). These relationships suggest that simultaneity windows are a good indicator of cross-modal temporal uncertainty.

Our experiments were designed to test the hypothesis that observers adjust their subjective criteria of cross-modal simultaneity based on their current temporal sensory uncertainty. Participants completed a typical audiovisual simultaneity judgment task: they indicated whether a light flash and a sound occurred simultaneously or not by pressing one of two buttons. To manipulate their temporal uncertainty, participants repeated this task in different audiovisual environments: a virtual walk through Midtown Manhattan, a virtual walk through a forest, or a lab environment with monotonous visual and auditory scenery. To ensure replicability, we tested two separate groups of participants in subsequent experiments. In the first experiment, participants repeated the task in three environments (city, nature, and lab) spread across several sessions. In the second experiment, they repeated the task in two environments (city and lab) administered within the same session. We derived estimates of participants’ sensory uncertainty and simultaneity criteria by fitting observer models to the simultaneity judgment data from each environment.

## Methods

### Participants

Forty participants (20 participants per experiment, see Online Supplementary Material (OSM) for simulation-based power analyses; 13 male, 17–37 years old, mean age 22 years) were recruited at Tufts University. Data of six additional participants were excluded; one experienced problems handling the button box, one pressed the same button in 93% of trials, and four had lapse rates above 15%. All participants reported normal or corrected-to-normal visual acuity and lack of any auditory, tactile, motor, and neurological impairments. The study was approved by the Institutional Review Board of Tufts University. Participants gave written informed consent prior to the beginning of the study.

### Apparatus and stimuli

**Fig. 2:**
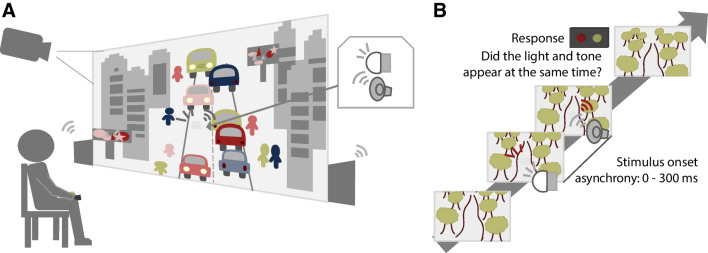
Setup and procedure. (**A**) Participants judged the temporal alignment of visual-auditory stimulus pairs presented via a hidden apparatus (inset) at the center of the wall in front of them. During the experiment, videos were projected onto the wall, immersing participants in different environments, for example, a walk through Midtown Manhattan filmed from the point of view of the observer. The accompanying sounds were presented through large speakers next to the screen. (**B**) In another environmental condition, the video showed a walk through a forest, again filmed from the perspective of the person taking the walk. The task-relevant audiovisual stimulus pairs were either presented simultaneously or with a temporal offset. Audiovisual stimulus pairs with varying orders and onset asynchronies were randomly interleaved. Participants judged the simultaneity of each stimulus pair by pressing one of two buttons

Participants sat at a table in a dark room facing a white wall 2 m in front of them. A white light-emitting diode (10 mm diameter) and a small speaker (40 mm, 4 Ohm, 3 Watt, www.adafruit.com) were mounted at eye level in the center of the wall (Fig. 2A). A white translucent cloth was secured over both items so that the apparatus appeared to blend in with the white wall. A projector (Epson VS260), mounted behind and above the participant, was used to project the videos on the wall (effective screen size 168 x 161 cm). Two large speakers (Klipsch R41PM) were positioned on both sides of the projected screen. Participants held a response box (Millikey SH-4, LabHackers, Halifax, Canada) in both hands so that their thumbs rested on the two outer buttons.

The task-relevant stimuli were audiovisual stimulus pairs presented via the apparatus in the center of the screen. The visual stimulus was a bright light (300 Lux) emitted by the LED. The auditory stimulus was a loud tone (50 dB), a 200-Hz square wave, presented through the small speaker. Each stimulus was 50 ms long. The two stimuli were presented in random order with a stimulus-onset asynchrony (SOA) of 0, 20, 50, 80, 100, 150, 200, 250, or 300 ms. The projected videos were a recording of a 15-min-long walk through the streets of Manhattan (Experiments 1 and 2), a recording of a 30-min-long walk through a forest with a creek (Experiment 1), a gray static image (Experiment 1), or a Mac OS screensaver showing colored dots moving in soft waves across a black background (Experiment 2). Both walks were filmed from the point of view of the person taking the walk and included sound recordings of the environment. The gray screen was paired with white noise, and the screensaver with an audio recording of rainfall. Videos and soundtracks were adjusted to be comparable in brightness and loudness.

The experiment was coded in Python and run via PsychoPy (version 2021.2.3, Peirce et al., 2019), which interfaced with a microcontroller (Arduino R3, Turin, Italy) to ensure precise timing of the auditory and visual stimuli. The raw data as well as our experimental and analysis scripts are publicly available via the Open Science Framework at: https://osf.io/q7fyg/?view_only=244522e40ab3447aa768a4576fc93df6.

### Procedure

In each trial, an audiovisual stimulus pair was presented. Participants indicated by button press whether they perceived the light and the sound as occurring simultaneously or non-simultaneously (Fig. 2B). The next trial started 1.5–2 s (uniform distribution) after the response had been registered. Trials without a response were aborted after 25 s (0.005% of trials) and no feedback was provided. Throughout the experiment, the video corresponding to the current environmental condition would be playing in an endless loop. Each of the 17 stimulus pairs was presented 30 times in randomized order, resulting in 510 trials per environmental condition. The trials were split into ten blocks and participants took on average of 25 min to complete all trials for one environmental condition. The order of the environmental conditions was randomized across participants. In Experiment 1 participants could choose whether they wanted to complete the three environmental conditions in one (no participants), two (18 participants), or three (two participants) sessions; in Experiment 2 both environmental conditions were tested in a single session.

### Analysis

The proportion of trials in which the stimulus pair was perceived as simultaneous was described as a function of the temporal offset between the stimuli. The model we fitted to the data assumes that the observer’s simultaneity judgment is based on the difference between the arrival times of the two stimuli in the relevant brain area, ${\Delta}_{{t}_{A}{t}_{V}}$ (independent-channels model ; Sternberg & Knoll, 1973). If both arrival times are distorted by Gaussian-distributed noise (Yarrow et al., 2022), the difference between the two arrival times is also a Gaussian-distributed random variable, centered on the physical temporal discrepancy of the two stimuli, ${\Delta }_{{t}_{A}{t}_{V}}\sim N({\text{SOA}},\sigma )$, where $\sigma =\sqrt{{\sigma }_{A}^{2}+{\sigma }_{V}^{2}}$ (Schneider & Bavelier, 2003; see OSM for an alternative model assuming exponentially distributed arrival times; this alternative model supports the same conclusions as the current model). The model further assumes that to generate a simultaneity judgment response, the observer compares the measured audiovisual temporal offset to two subjectively set criteria: ${C}_{AV}$, for trials in which the auditory stimulus arrived before the visual one, and ${C}_{VA}$, for trials in which the auditory stimulus arrived after the visual one. The observer reports that the stimuli were simultaneous if the perceived temporal offset remains below the corresponding boundary. Thus, the probability to perceive the stimuli as simultaneous corresponds to the probability of measuring an audiovisual temporal offset smaller than the relevant boundary, ${\text{P}}\left({s}_{{\text{simultaneous}}}\right)={\text{P}}\left({\Delta }_{{t}_{A}{t}_{V}}<{C}_{AV}\right)=\Phi ({C}_{AV};{\text{SOA}}, \sigma )$ if the auditory stimulus arrived first and ${\text{P}}\left({s}_{{\text{simultaneous}}}\right)={\text{P}}\left({\Delta }_{{t}_{A}{t}_{V}}<{C}_{VA}\right)=\Phi ({C}_{VA};{\text{SOA}}, \sigma )$ if the visual stimulus arrived first. If we code the measured temporal offsets on a continuous scale, i.e., if ${\Delta }_{{t}_{A}{t}_{V}}$ corresponds to negative values if the auditory stimulus arrived first and to positive values if the visual stimulus arrived first, we can express the probability that the observer judges the stimuli as simultaneous with one term ${\text{P}}\left(-{C}_{AV}<{\Delta }_{{t}_{A}{t}_{V}}<{C}_{VA}\right)=\Phi ({C}_{VA};{\text{SOA}}, \sigma )-\Phi (-{C}_{AV};-{\text{SOA}}, \sigma )$. Finally, we assumed that the observer lapses with rate $\lambda$ and thus presses the button corresponding to “simultaneous” with probability $P\left({r}_{{\text{simultaneous}}}\right)=0.5\lambda +(1-\lambda )(\Phi \left({C}_{VA};{\text{SOA}}, \sigma \right)-\Phi \left(-{C}_{AV};-{\text{SOA}}, \sigma \right))$.

We fit the model to each participant’s responses by finding the set of parameters $\{\sigma ,{C}_{AV},{C}_{VA},\lambda \}$ that minimized the negative log-likelihood. To avoid being stuck in local minima, we obtained start parameters using a brute force grid search before running the optimization algorithm. Separate parameter estimates were generated for each participant and environmental condition. We additionally fitted a model variant that assumed no differences between environmental conditions, i.e., parameter estimates in this variant were derived based on data from all conditions. We compared the fit (quantified as AIC values) of this reduced environment-independent model to that of the main model to check for participant-specific effects of environmental condition.

To test our main hypothesis that the subjective criterion is adjusted based on the observer’s current temporal uncertainty, we fit a linear mixed model with the criterion as the dependent variable. The uncertainty parameter $\sigma$, the type of criterion (auditory-first or visual-first), and the environmental condition were included as predictors, and we estimated participant-level intercepts. To check whether the environmental condition had a systematic effect on participants’ temporal uncertainty, we fit a mixed model with uncertainty parameter $\sigma$ as the dependent variable and environmental condition as predictor.

## Results

**Fig. 3:**
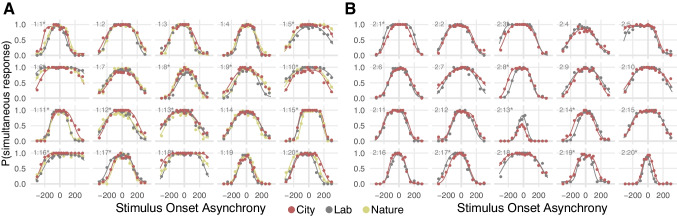
Audiovisual simultaneity judgment data and model fits. The probability to perceive an audiovisual stimulus pair as simultaneous is shown as a function of the stimulus-onset asynchrony of the two stimuli (negative values indicate “auditory first”-stimulus pairs, positive values indicate “visual first”-stimulus pairs). Observed data (markers) and model predictions (lines) are shown for each of the different virtual environments the experiment was conducted in (red: walk through Midtown Manhattan; gray: monotonous lab environment; yellowish green: walk through a forest). Each panel shows data from one participant (identifiers in the upper left corners; a star next to the identifier indicates that the model assuming participant-specific changes in uncertainty and criterion across environments fit the data better than an environment-independent model), (**A**) 20 participants for Experiment 1, in which three different environments were administered across multiple sessions, and (**B**) 20 different participants for Experiment 2 in which two environments were tested in the same session

**Fig. 4:**
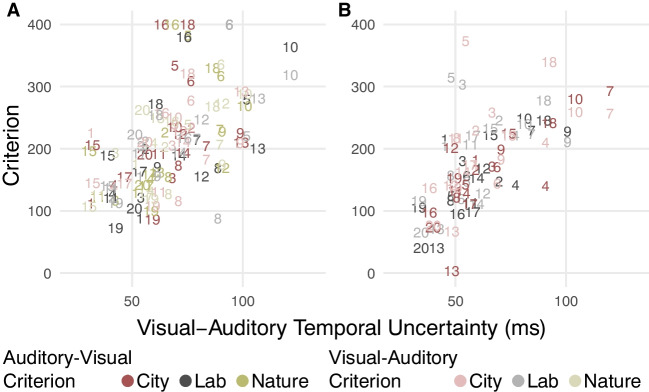
Subjective simultaneity criteria as a function of audiovisual temporal uncertainty. The subjective criterion determines which measured auditory-visual (light hues) or visual-auditory (dark hues) temporal offset marks the boundary between measured stimulus offsets categorized as stemming from simultaneous stimuli and those categorized as stemming from non-simultaneously presented stimuli. Each participant’s boundaries in the different environmental conditions are shown as a function of the participant’s estimated audiovisual temporal uncertainty in that condition (red: city, gray: lab, yellowish green: nature). Twenty participants completed each of the two experiments, Experiment 1 (**A**) and Experiment 2 (**B**). To facilitate a comparison between the psychometric curves typically used to show performance in the simultaneity judgment task and the estimated parameters, markers correspond to the participant identifiers in Fig. 3

Participants’ responses in both experiments were well described by the independent-channels model (Fig. 3). Participants’ audiovisual temporal uncertainty, quantified as parameter $\sigma$, significantly predicted their audiovisual simultaneity criterion (Exp. 1: χ^2^(1) = 14.57, *p* < 0.001; Exp. 2: χ^2^(1) = 15.88, *p* < 0.001; Fig. 4). In Experiment 2, an additional significant effect of criterion type emerged: participants used a more liberal criterion when judging auditory-visual offsets compared to judging visual-auditory offsets (χ^2^(1) = 16.39, *p* < 0.001). No significant group effects of environment on the criterion or temporal uncertainty emerged. Model comparisons indicated participant-specific effects of environment for 14 out of 20 participants in Experiment 1 and eight out of 20 participants in Experiment 2; a model using the same parameters across virtual environments fit the data of these participants significantly worse (${\Delta }_{AIC}>10$).

## Discussion

Here, we investigated the relationship between the two determinants of audiovisual simultaneity judgments: (1) temporal uncertainty and (2) decision criteria. These criteria are subjectively set internal boundaries that account for the noisiness of perceptual measurements. Measured temporal offsets small enough to fall below the criterion will be judged as originating from simultaneously presented stimuli; larger offsets will lead to the conclusion that the stimuli were presented asynchronously. We tested the hypothesis that observers follow the performance-enhancing strategy of adjusting their subjective criteria of cross-modal simultaneity based on their current temporal uncertainty. Participants completed an audiovisual simultaneity judgment task while being immersed in different virtual environments, which unsystematically influenced their temporal uncertainty. As hypothesized, participants’ decision boundaries in an environment were predicted by their temporal uncertainty in that condition. Hence, participants used a flexibly updated estimate of their own audiovisual temporal uncertainty to establish subjective criteria of simultaneity for the current environment. This finding further implies that, under typical circumstances, audiovisual simultaneity windows directly and indirectly reflect an observer’s cross-modal temporal uncertainty.

Our participants performed the simultaneity judgment task in different environments – on a virtual walk through Midtown, on a walk through the woods, or in a monotonous lab setting. These environments affected participants’ sensory uncertainty. For most participants, performance was best captured by a model assuming different perceptual parameters for different environments, but the direction of the effects of environment was unsystematic across participants. Most likely no systematic effect emerged because a monotonous environment can have very different effects on participants’ attention towards the target stimuli, a major determinant of sensory uncertainty (Badde, Navarro, et al., 2020b; Vercillo & Gori, 2015). In addition, participants’ sensory uncertainty might have varied with the time point of testing given that the effects of environmental condition were more frequent in the first than in the second experiment, i.e., when testing was stretched out across several sessions. Importantly for our research question, only participants’ environment-specific temporal uncertainty, not the environmental condition itself, significantly predicted the decision criteria they used in an environment. Hence, the influence of the different environments on temporal uncertainty must have been registered and taken into account during the decision process.

Here, we show that observers flexibly account for their sensory uncertainty when establishing a subjective decision criterion of perceived audiovisual simultaneity. This is a performance-enhancing strategy, yet nevertheless remarkable in that it requires a flexibly updated estimate of one’s own sensory uncertainty. Similar to our finding, observers take their sensory uncertainty into account when setting subjective criteria in tasks in which they indicate their confidence in their own perceptual performance (Denison et al., 2018; Fleming & Daw, 2017; Locke et al., 2022; Mamassian, 2011). And observers optimally account for their sensory (Badde, Navarro, et al., 2020b; Ernst & Banks, 2002; Hong et al., 2021; Körding et al., 2007; Trommershäuser et al., 2011) and motor (Faisal & Wolpert, 2009; Hudson et al., 2010; Zhang et al., 2013) uncertainty in a multitude of perceptual tasks that do not contain a subjective component. Theoretical models suggest that sensory uncertainty is encoded in the population-level responses of neurons in sensory cortices (Ma et al., 2006; Ma & Jazayeri, 2014), and newer fMRI methods decode sensory uncertainty in the BOLD signals from early sensory cortices (Van Bergen et al., 2015). Thus, there is ample evidence that the brain has a representation of sensory uncertainty that is updated based on the sensory context. Yet, the results obtained here and in many other studies leave it open as to whether the observers’ estimates of their own sensory uncertainty are correct. 

Observers tolerated larger temporal offsets between the stimuli when their temporal uncertainty and thus the to-be-expected noisiness of their measurements was higher. This behavior accounts for the noisiness of perceptual measurements in a flexible manner and intuitively improves participants’ perceptual decisions. However, we can only speculate about how far our participants behaved “optimally”, i.e., adjusted their judgments identically to an ideal observer who minimizes a specific cost function. An ideal observer relying on Bayesian principles would base the simultaneity judgments on the posterior probability that both measurements, the auditory and the visual one, originated from a common event, i.e., have a common cause (Hong et al., 2023; McGovern et al., 2016). Yet, we repeatedly found that observers employed a suboptimal strategy when judging the spatial (and temporal) alignment of cross-modal stimulus pairs. Instead of directly relying on the posterior probability of a common cause, they compared their perceptual estimates, which in turn were derived based on a causal inference process, to a decision boundary (Badde, Navarro, et al., 2020b; Hong et al., 2021, 2022, 2023). Consistently, in the temporal domain, it has been reported that a model with causal-inference-based decision boundaries fits audiovisual simultaneity judgment data across a variety of speech stimuli better than a model-free approach of fitting a symmetric function to the data (Magnotti et al., 2013). Yet, subsequent studies reported that an independent-channels model in which the boundaries are free parameters, similar to the one used here, fits asymmetric data better and that the temporal causal inference-based criterion model is not identifiable (García-Pérez & Alcalá-Quintana, 2015b). Indeed, in our previous studies, we were only able to distinguish between different causal inference models because participants completed a spatial estimation task in addition to making binary judgments of spatial (mis-)alignment. Hence, whether participants acted optimally or just similar to ideal observers is unlikely to be decided based on classical simultaneity judgment data.

Audiovisual simultaneity windows are often treated as a proxy of multisensory integration (Donohue et al., 2012; Habets et al., 2017; Stevenson et al., 2014; Wallace & Stevenson, 2014), an approach that has come under scrutiny because of the role of subjective decision criteria in the simultaneity judgment task (García-Pérez & Alcalá-Quintana, 2015a; Linares & Holcombe, 2014; Petrini et al., 2020; Yarrow, 2018; Yarrow et al., 2023). Our results reveal that these subjective criteria mirror temporal uncertainty within and across participants (see OSM), and thus that simultaneity windows might directly as well as indirectly (through the criterion) reflect audiovisual temporal uncertainty. In turn, audiovisual temporal uncertainty is an important determinant of audiovisual integration; sensory uncertainty strongly affects whether cross-modal signals are perceived as originating from a common cause and should be integrated (Badde et al., 2023; Badde, Navarro, et al., 2020b; Hong et al., 2021, 2022; Körding et al., 2007). Hence, the relationship between cross-modal simultaneity windows and multisensory integration might go back to the influence of sensory uncertainty on both. Nevertheless, other factors such as an observer’s a priori assumptions about the shared origin of cross-modal signals influence multisensory integration (Badde et al., 2023, Badde, Navarro, et al., 2020b; Körding et al., 2007), and cross-modal temporal perception is permanently shaped by sensory experience during early development (Badde, Ley, et al., 2020a; Chen et al., 2017). So, whereas our data support and extend the relation between simultaneity windows and multisensory integration, these factors remain separate processes (Harrar et al., 2017) influenced but not exclusively determined by sensory uncertainty.

The here-reported estimates of audiovisual temporal uncertainty correspond to the expected measurement error, assuming this error is Gaussian-distributed (Schneider & Bavelier, 2003). This version of the independent-channels model describes the observed data, which vary considerably across participants, exceptionally well (Fig. 3). Yet, the derivation of the model is based on Gaussian-distributed arrival times (see Methods), and thus a non-zero probability is assigned to the impossible situation that a stimulus (auditory or visual) might be registered too early in the brain. This conceptual problem is avoided by approaches that use exponential rather than Gaussian distributions to model the arrival times of each stimulus (García-Pérez & Alcalá-Quintana, 2012; Petrini et al., 2020). Fitting such a model to our data leads to the same conclusion that subjective decision criteria are based on temporal uncertainty (OSM). However, the exponential model did not provide a better fit of the data (OSM), despite (or because) the higher number of free parameters. Moreover, even by combining the audiovisual exponential decay parameters that represent temporal uncertainty in the exponential arrival times model into a single measure, we would not obtain a measure that translates directly into the average temporal error, whereas the standard deviation of the Gaussian distribution does so. Thus, the simpler Gaussian-based model provided a better and more accessible description of the data in this study.

In conclusion, this study reveals that naïve observers flexibly adjust their subjective criteria of audiovisual simultaneity based on sensory uncertainty, suggesting that under typical circumstances, simultaneity windows directly and indirectly reflect audiovisual temporal uncertainty.

## Supplementary Information

Below is the link to the electronic supplementary material.

Supplementary file1 (PDF 755 KB)

## Data Availability

Data as well as experimental and analysis scripts are publicly available via the Open Science Framework (https://osf.io/q7fyg/?view_only=244522e40ab3447aa768a4576fc93df6).
