# Supplementary material for: Audiovisual simultaneity windows reflect temporal sensory uncertainty
Source: Psychon Bull Rev. 2024 Feb 22;31(5):2170–9. doi: 10.3758/s13423-024-02478-4 (PMC11543760; doi:10.3758/s13423-024-02478-4)
Supplement: Supplementary file 1 — Supplementary file1 (PDF 755 KB) [file 13423_2024_2478_MOESM1_ESM.pdf]

Supplementary Information

Simulation-Based Power Analysis

Our design required a linear mixed model, predicting participants' criteria from their temporal uncertainty as well as the type of criterion and the virtual environment they were in when the data was collected. Each participant completed the task in three (or two in Expt. 2) environments. For each of these environments an estimate of sensory uncertainty as well as two decision criteria (one for auditory before visual and one for visual before auditory decisions) were derived. There is no closed form solution for a power analysis, thus, we used simulations to establish a reasonable sample size. The simulations were run for a wide range of fixed effects, ranging from 0.6 to 3 for the effect of temporal uncertainty, which itself ranged from 20 to more than 100 ms, and from 0 to 20 for the contrast-coded categorical predictors. We assumed an error standard deviation of 50 ms and the same for the standard deviation of the random intercept. Values larger than that would induced a variability in the criteria that would have exceeded the range of possible values for the criterion. Each combination was simulated 5000 times. The results of the power analysis are shown below (**Fig. 1**). For all but the lowest tested beta value the power was far above 80% for a sample size of 20.

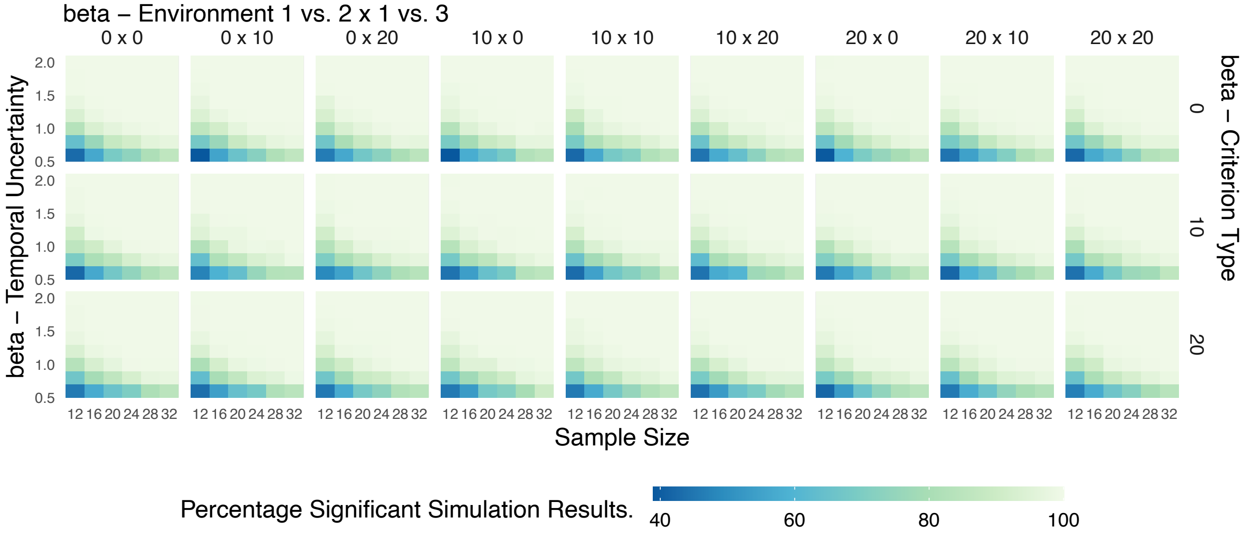

Fig. S1. Simulation-Based Power Analysis. The percentage of simulations leading to a significant result for our main effect of interest, the fixed effect of sensory uncertainty on participants' decision criteria, is color-coded. Simulations were run for different sample sizes (x-axis) and a range of values for the effect of temporal uncertainty (y-axis), virtual environment (3 levels split into two comparisons; columns) and the type of criterion (auditory-before-visual and visual-before-auditory; rows).

Exponentially-Distributed Arrival Times

Model

The model described in this section is identical to the main model in that it assumes that the observer's simultaneity judgment is based on the difference between the arrival times of the two stimuli in the decision-making brain area, $\Delta_{t_A t_V}$, (1). The here described model assumes that the arrival times of the auditory and visual signals in the relevant brain area are exponentially distributed, with decay parameters λ_A and λ_V and a constant modality-specific delay, τ_A and τ_V. The probability distribution of the difference between the two arrival times is a composite function, its value depends on the perceived order of the two modalities,

$$f\left(\Delta_{t_A t_V}; \mathrm{SOA}, \lambda_A, \lambda_V, \tau_A, \tau_V\right) =$$

$$\begin{cases} \frac{\lambda_A \lambda_V}{\lambda_A + \lambda_V} \exp\left(\lambda_V \left(\Delta_{t_A t_V} - SOA - (\tau_A - \tau_V)\right)\right), \text{if } \Delta_{t_A t_V} \leq \mathrm{SOA} + (\tau_A - \tau_V) \\ \frac{\lambda_A \lambda_V}{\lambda_A + \lambda_V} \exp\left(-\lambda_A \left(\Delta_{t_A t_V} - SOA - (\tau_A - \tau_V)\right)\right), \text{if } \Delta_{t_A t_V} > \mathrm{SOA} + (\tau_A - \tau_V) \end{cases} \quad (2,3).$$

We still assume that the observer judges a stimulus pair as simultaneous if the measured temporal offset lies in between the subjective criteria of simultaneity, C_{AV} and C_{VA}. Thus, the probability that the observer intends to respond 'simultaneous' equals the area under the measurement distribution between these two boundaries (**Fig. 2A**), and again they might lapse with probability λ, $P(r_{\mathrm{simultaneous}}) = 0.5\lambda + (1-\lambda)(\mathrm{F}(C_{VA}; \mathrm{SOA}, \sigma) - \mathrm{F}(-C_{AV}; -\mathrm{SOA}, \sigma))$,

$$F(x; \mathrm{SOA}, \lambda_A, \lambda_V, \tau_A, \tau_V) =$$

$$\begin{cases} \frac{\lambda_A \lambda_V}{\lambda_A + \lambda_V} \exp\left(\lambda_V\left(x - SOA - (\tau_A - \tau_V)\right)\right), \text{if } x \leq \mathrm{SOA} + (\tau_A - \tau_V) \\ 1 - \frac{\lambda_A \lambda_V}{\lambda_A + \lambda_V} \exp\left(-\lambda_A\left(x - SOA - (\tau_A - \tau_V)\right)\right), \text{if } x > \mathrm{SOA} + (\tau_A - \tau_V) \end{cases} \quad (2,3; \textbf{Fig. 2B})$$

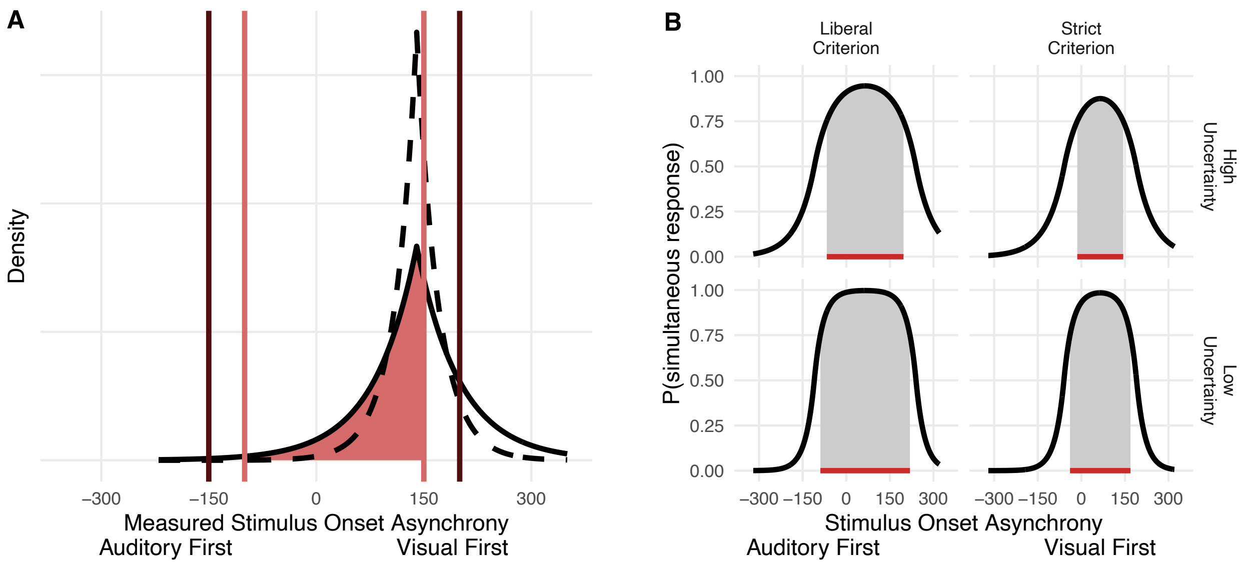

Fig. S2. Independent channels model given exponentially distributed arrival times. (A) Measurement distributions of the perceived temporal offset between the auditory and the visual stimulus given exponentially distributed arrival times. Here, the fixed, modality-specific delays

equal $\tau_A = 20$ ms and $\tau_V = 60$ ms. The standard deviation, $1/\lambda$, used to generate the high uncertainty distribution (solid line) equals 60 ms, the standard deviation for the low uncertainty curve (dashed line) equals 30 ms. Here, uncertainty is constant across modalities, however, the model allows for modality-specific temporal uncertainty. The vertical lines show the liberal (burgundy) and strict (salmon) criteria used to generate the psychometric functions in (B). As for the Gaussian-based independent channels model, the simultaneity windows depend on temporal uncertainty as well as the decision criteria.

We fit this model to each participant's responses by finding the set of parameters $\{\lambda_A, \lambda_V, \tau = \tau_A - \tau_V, C_{AV}, C_{VA}, \lambda\}$ that minimized the negative log-likelihood. C_{AV}, C_{VA}, and τ trade off against each other. Thus, researchers often fix $C_{AV} = -C_{VA}$ (3). However, doing so would have complicated the comparison to the model used in the main text. To avoid being stuck in local minima, we obtained start parameters using a brute force grid search before running the optimization algorithm. Separate parameter estimates were generated for each participant and environmental condition.

To test our main hypothesis that the subjective criterion is adjusted based on the observer's current temporal uncertainty, we fit two linear mixed models with the criterion as the dependent variable, one model used auditory uncertainty as predictor, the other one visual uncertainty. In addition, the type of criterion (auditory-first or visual-first), and the environmental condition were included as predictors, and we estimated participant-level intercepts.

Results

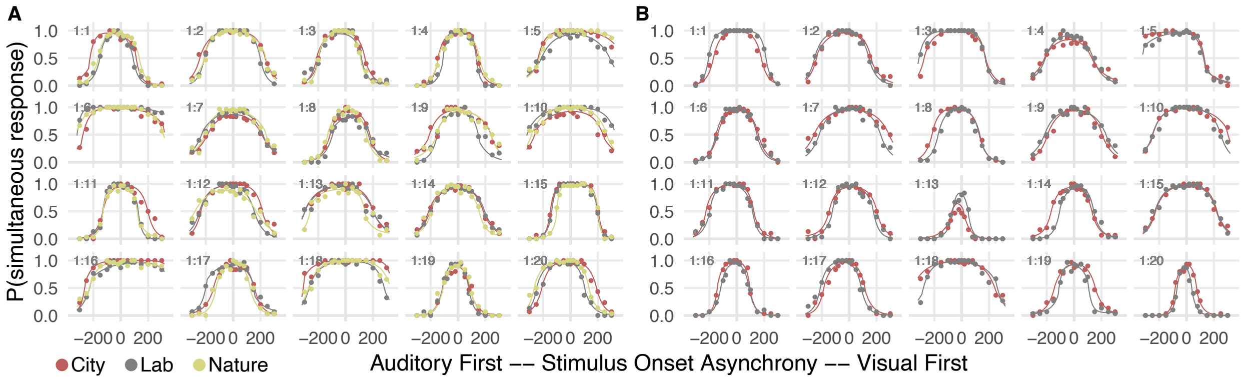

Fig. S3. Audiovisual simultaneity judgment data and model fits. The probability to perceive an audiovisual stimulus pair as simultaneous is shown as a function of the stimulus onset asynchrony of the two stimuli (negative values indicate 'auditory first'-stimulus pairs, positive values indicate 'visual first'-stimulus pairs). Observed data (markers) and model predictions (lines) are shown for each of the different virtual environments the experiment was conducted in (red: walk through Midtown Manhattan; grey: monotonous lab environment; yellowish green: walk through a forest). Each panel shows data of one participant (identifiers in the upper left corners), (A) 20 participants for Expt. 1, in which three different environments were administered across multiple sessions and (B) 20 different participants for Expt. 2 in which two environments were tested in the same session.

Participants' responses in both experiments were described reasonably well by the independent channels model (**Fig. 3**). However, the fit was slightly worse (the negative log likelihood was higher) than that of the Gaussian-based model. Notably, Participants' audiovisual temporal uncertainty, quantified as $1/\lambda_A$ and $1/\lambda_V$, typically predicted their audiovisual simultaneity criterion (Expt. 1, visual: $\chi^2(1)=4.78$, $p=0.029$; Expt. 1, auditory: $\chi^2(1)=0.41$, $p=0.522$; Expt. 2, visual: $\chi^2(1)=5.81$, p=0.016; Expt. 2, auditory: $\chi^2(1)=8.58$, $p=0.003$; **Fig. 4**).

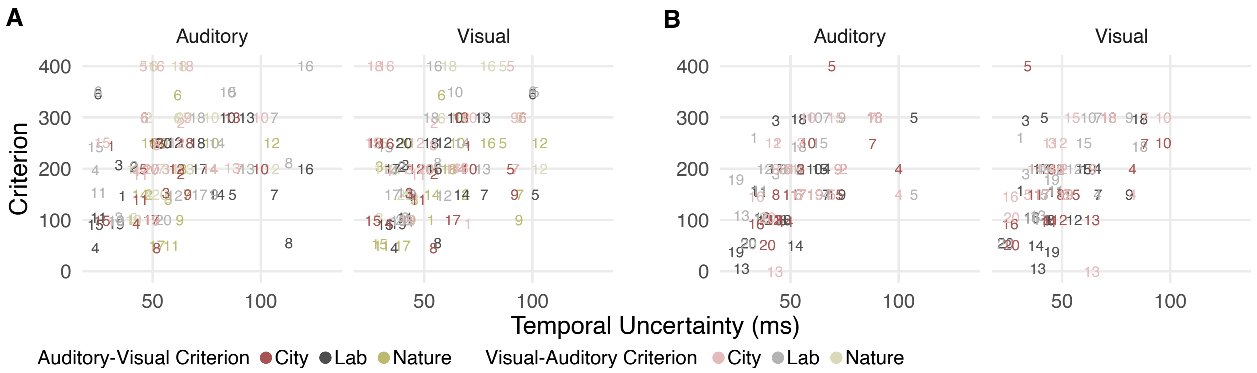

Fig. S4. Subjective simultaneity criteria as a function of auditory and visual temporal uncertainty. The subjective criterion determines which measured auditory-visual (light hues) or visual-auditory (dark hues) temporal offset marks the boundary between measured stimulus pairs categorized as simultaneous or those categorized as not simultaneous. Each participant's boundaries in the different environmental conditions are shown as a function of the participant's estimated auditory and visual temporal uncertainty in that condition (red: city, grey: lab, yellowish green: nature). Twenty participants completed each of the two experiments, Expt. 1 (A) and Expt. 2 (B). To facilitate a comparison between the psychometric curves typically used to show performance in the simultaneity judgment task and the estimated parameters, markers correspond to the participant identifiers in Fig. S3.

Effects of Absolute Uncertainty

Our main analysis shows that observers adjusted their audiovisual simultaneity criteria based on their temporal uncertainty. However, the mixed model we used to analyze the parameter estimates does not differentiate within- from between-subject effects. In theory, observers might adjust the criterion based on their relative temporal uncertainty in the different environments but not based on their absolute uncertainty, i.e., uncertainty might predict the criterion within but not across participants. To check for this possibility, we averaged each observer's uncertainty and criterion values and conducted a linear regression. Uncertainty predicted the criterion for both experiments (Expt. 1, $t(18)=2.58$, $p=0.001$; Expt. 2, $t(18)=4.90$, $p<0.001$), confirming that observers adjusted the criteria based on absolute uncertainty.

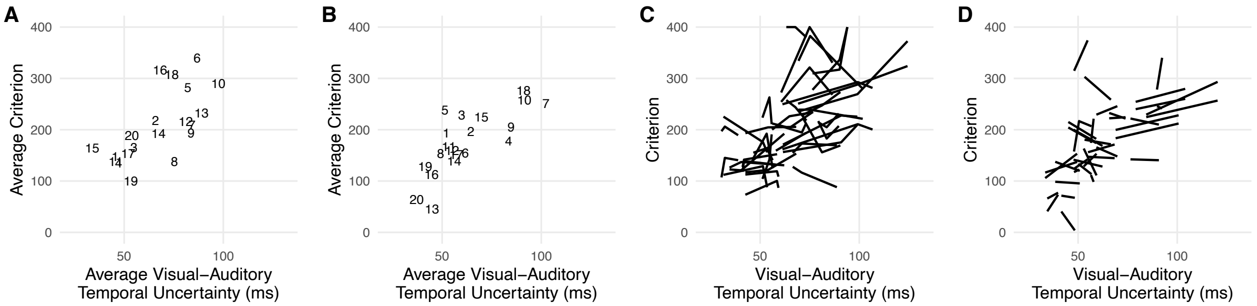

Fig. S5. Within- and between-subject effects of temporal uncertainty on the subjective criteria of simultaneity. Participant-level averages of the criterion values as a function of participant-level averages of temporal uncertainty in Expt. 1 (A) and Expt. 2 (B). Participant-level effects emphasized by replotting Fig. 4. The lines show each participant's criteria as a function of their temporal uncertainty in the corresponding session of Expt. 1 (C) and Expt. 2 (D).

References

1. Sternberg S, Knoll RS. The perception of temporal order: Fundamental issues and a general model. In: Attention and Performance IV. 1973. p. 629–85.

2. García-Pérez MA, Alcalá-Quintana R. On the discrepant results in synchrony judgment and temporal-order judgment tasks: a quantitative model. Psychon Bull Rev. 2012 Oct;19(5):820–46.

3. García-Pérez MA, Alcalá-Quintana R. Converging evidence that common timing processes underlie temporal-order and simultaneity judgments: a model-based analysis. Atten Percept Psychophys. 2015 Jul;77(5):1750–66.
